# ZIP13: A Study of *Drosophila* Offers an Alternative Explanation for the Corresponding Human Disease

**DOI:** 10.3389/fgene.2017.00234

**Published:** 2018-01-31

**Authors:** Guiran Xiao, Bing Zhou

**Affiliations:** ^1^School of Food Science and Engineering, Hefei University of Technology, Hefei, China; ^2^State Key Laboratory of Membrane Biology, School of Life Sciences, Tsinghua University, Beijing, China

**Keywords:** Drosophila, iron, zinc, ZIP13, SCD-EDS

## Abstract

The fruit fly *Drosophila melanogaster* has become an important model organism to investigate metal homeostasis and human diseases. Previously we identified dZIP13 (CG7816), a member of the ZIP transporter family (SLC39A) and presumably a zinc importer, is in fact physiologically primarily responsible to move iron from the cytosol into the secretory compartments in the fly. This review will discuss the implication of this finding for the etiology of Spondylocheirodysplasia-Ehlers-Danlos Syndrome (SCD–EDS), a human disease defective in ZIP13. We propose an entirely different model in that lack of iron in the secretory compartment may underlie SCD-EDS. Altogether three different working models are discussed, supported by relevant findings made in different studies, with uncertainties, and questions remained to be solved. We speculate that the distinct ZIP13 sequence features, different from those of all other ZIP family members, may confer it special transport properties.

## Introduction: spondylocheirodysplasia-ehlers-danlos syndrome and ZIP13

Ehlers-Danlos syndrome (EDS) is a group of clinically and genetically heterogeneous disorders with defects in connective tissue characterized by skin hyperelasticity, tissue fragility, poor wound healing, and joint hypermobility (Yeowell and Pinnell, 1993; Beighton et al., 1998; Bowen et al., 2017; Brady et al., 2017). In the Villefranche Nosology, EDS is divided into six subtypes: the classical, hypermobile, vascular, kyphoscoliotic, arthrochalasis, and dermatosparaxis subtypes (Bowen et al., 2017; Brady et al., 2017). The pathogenesis of these diseases was not defined until recently. Defects of fibrillary collagens or collagen-modifying enzymes had been identified in all subtypes except the hypermobile subtype (Bowen et al., 2017; Zhang et al., 2017).

**Figure 1:**
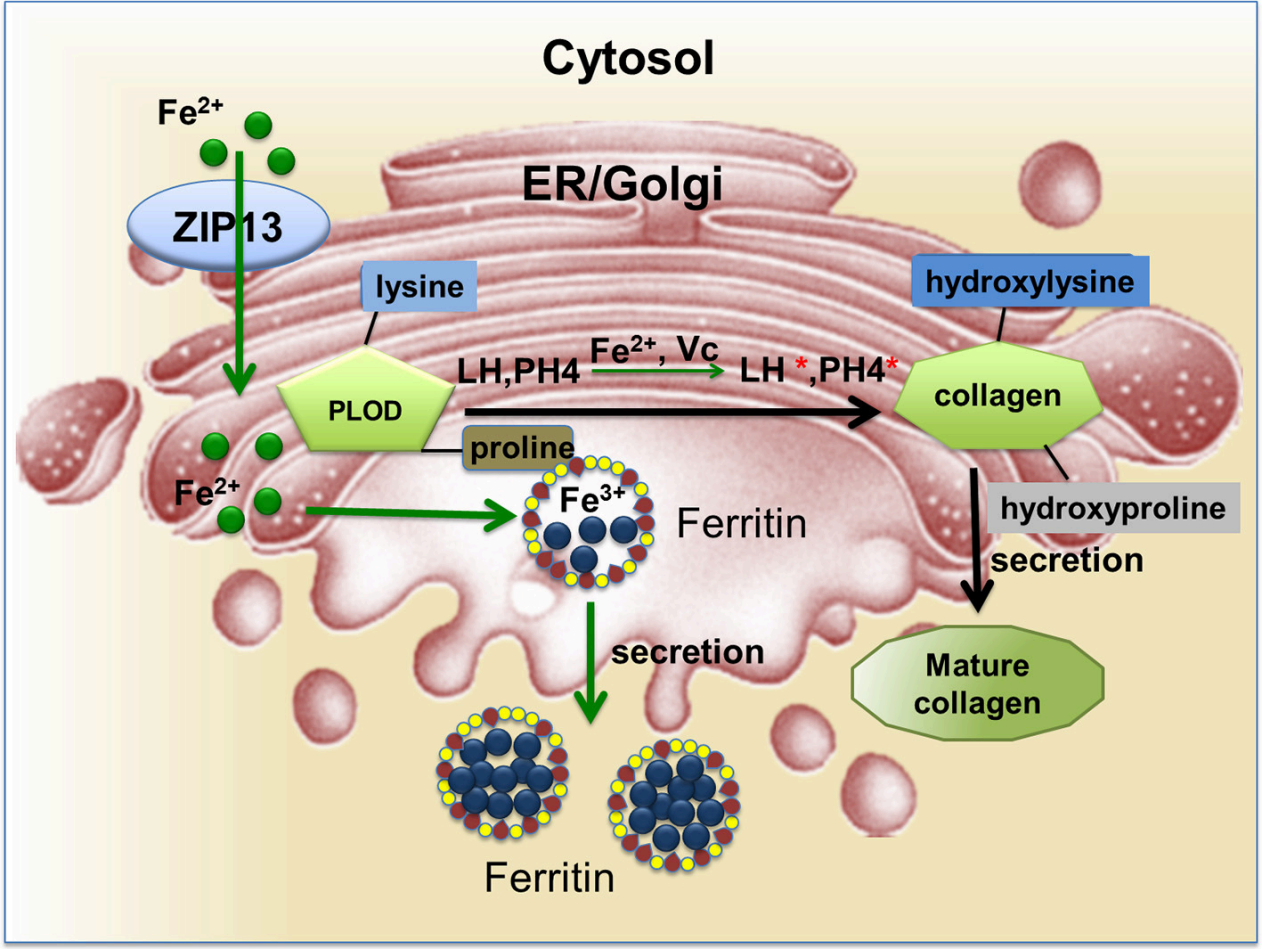
LH and PH4 are critical enzymes for collagen hydroxylation during collagen maturation. The reactions require iron and ascorbate as cofactors. In this figure, ZIP13 is proposed as the iron exporter localized on the ER/Golgi and responsible for iron transporting from the cytosol into the secretory pathway. These iron ions will be utilized by proteins such as ferritin (in the fly and likely some other insects with secretion type of ferritin) and LH.

SCD–EDS is a very rare autosomal recessive disease caused by mutations in the hZIP13 gene (Fukada et al., 2008; Giunta et al., 2008; Calap-Quintana et al., 2017). An increased ratio of total urinary pyridinolines, lysylpyridinoline/hydroxylysylpyridinoline (LP/HP) was found in patients (Calap-Quintana et al., 2017), but the total amount of pyridinolines (LP plus HP) was not affected (Calap-Quintana et al., 2017). This indicates that the formation of LP was increased while HP was decreased. Same situation was observed in another disease, Ehlers-Danlos type VI (EDS VI), which shares several clinical signs with SCD–EDS (Steinmann et al., 1995). The hydroxylation of lysyl and prolyl residues in collagens was measured *in vivo* and *in vitro*. Lysyl under-hydroxylation and prolyl under-hydroxylation are not confined to specific residues and occur along the whole molecule (Giunta et al., 2008). In normal people, these hydroxylation processes are catalyzed by lysyl hydroxylase (LH) and prolyl 4-hydroxylase (PH4) (Figure 1; Myllyharju, 2008). Notably, EDS VI is caused by mutations in the PLOD1 gene, which encodes lysyl hydroxylase (LH1) (Yeowell and Walker, 2000; Yeowell et al., 2000). These indicate SCD–EDS is closely related to collagen under-hydroxylation.

After excluding PLOD1, PLOD2, and PLOD3, ZIP13 mutations were finally identified as the culprit in SCD–EDS patients (Giunta et al., 2008). Bin et al. demonstrated that both the identified human mutation ZIP13^G64D^ and ZIP13^Δ*FLA*^ result in rapid degradation of the mutant protein in cultured primary cultured fibroblasts, and an imbalance of intracellular zinc homeostasis (Bin et al., 2014). Importantly, proteasome inhibitor MG132 treatment increased the total ZIP13 levels and restored the impaired intracellular zinc homeostasis of the SCD–EDS patient cells (Bin et al., 2014).

ZIP13 belongs to the ZIP (Slc39A) transporter family (To simplify, all Slc39A13 of various organisms are noted as “ZIP13” in this review). Most members of ZIP family transport zinc, but a few have also been found to transport other metals including iron. Most ZIP proteins have eight transmembrane domains (TMs) with a His-rich domain between TM3 and TM4, and hydrophilic residues in TM5 which may determine metal specificity (Taylor et al., 2007; Fukada and Kambe, 2011). The C- and N-terminal of ZIP proteins both face the extra cytoplasmic space (Lichten and Cousins, 2009; Jeong and Eide, 2013; Kambe et al., 2014). Based on amino acid sequence similarity ZIP family can be divided into four subfamilies: ZIPI, ZIPII, GufA, and LZT (Taylor and Nicholson, 2003; Fukada and Kambe, 2011).

Biochemical characterization of human ZIP13 protein by Bin et al. indicated that ZIP13 is located mainly in the Golgi apparatus, with both its N and C termini end in the luminal side (Bin et al., 2011). Zip13 is classified to be a member of the ZIP LZT subfamily, which is characterized by eight putative TMs and a unique hydrophilic region. ZIP13 additionally possesses some domains that are not found in other LZT family members (Taylor and Nicholson, 2003; Bin et al., 2011; Fukada and Kambe, 2011).

One thing puzzling is that ZIP13 lacks characteristic His residues at the N-terminal and in the region between TM3 and TM4 (Bin et al., 2011). It is believed that these regions in other ZIPs can bind zinc ions (Taylor et al., 2007). Potocki et al. evaluated metal ion binding properties of the cysteine-rich N-terminal domain fragment of the ZIP13 zinc transporter (MPGCPCPGCG–NH_2_) and the results indicate that the binding ability of this fragment changes in the series Bi^3+^ >> Cd^2+^ > Zn^2+^ > Ni^2+^ (Potocki et al., 2011). This region of ZIP13, however, is not evolutionarily conserved in sequence. For example, in the *chicken* ZIP13, there is not this N-domain with several nearby “C”s or alternatively “H”s.

Structural information of ZIP13 is not available. Nevertheless, crystal structure of the extracellular domain (ECD) of mammalian ZIP4 has been solved by Zhang et al., demonstrating that ZIP4-ECD is a crucial regulatory domain for optimal zinc transport (Zhang et al., 2016). Very recently the same group solved the crystal structures of a prokaryotic ZIP from *Bordetella bronchiseptica* (BbZIP), indicating that two metal ions were trapped halfway through the membrane, unexpectedly forming a binuclear metal center. This work provides the first structural framework for understanding the metal transport mechanism of the ZIPs (Zhang et al., 2017). But at this stage, it is not still clear how zinc is moved across the membrane, and the relationship between the two bound metal ions (Zhang et al., 2017). For ZIP13, the critical question is: how mutations in ZIP13, a likely zinc transporter, could lead to collagen under-hydroxylation? One further finding worthy of noting is that despite collagen under-hydroxylation in SCD–EDS patients, *in vitro* enzymatic assays revealed that LH and PH4 activities were normal in patients' fibroblasts (Giunta et al., 2008). It appears that certain things might differ under *in vivo* and *in vitro* conditions so that hydroxylation could happen *in vitro* but not so *in vivo*.

## Initial evidences of dZIP13 involving in iron metabolism

ZIP family members are key zinc importers in mammalian organisms. Studying zinc homeostasis in *Drosophila* is a relatively recent event, but large strides have already been made (Richards and Burke, 2016; Xiao and Zhou, 2016), thanks to several distinctive advantages of this model organism *Drosophila* genome encodes at least eight putative ZIP proteins (Lye et al., 2012; Qin et al., 2013). Bioinformatics indicates that the protein encoded by *Drosophila* CG7816 (ZIP99c) shares the highest overall homology with human ZIP13 (we named it dZIP13 accordingly based on sequence and functions; Xiao et al., 2014). Similar to hZIP13, dZIP13 also has several typical features of LZT family members (Jeong and Eide, 2013), including the predicted eight TMs, particularly amphipathic TM4 and TM5, the luminal location of both N and C termini and the highly conserved potential metalloprotease motif (HEXXH, where X is any amino acid) within TM5 (Jeong and Eide, 2013; Xiao et al., 2014). Notably, the single His residue of hZIP13 in a generally histidine-rich region (2–14 His) between TM3 and TM4, is also found in dZIP13 (Xiao et al., 2014). Genetic evidence suggested that human ZIP13 may function similarly to dZIP13 in *Drosophila* (Xiao et al., 2014), corroborating this protein is the hZIP13 ortholog in *Drosophila*.

Unexpectedly, we discovered in dZIP13 knockdown (dZIP13–RNAi) flies a lower total amount of iron, but relatively normal amount of zinc, in their bodies (Xiao et al., 2014). This indicates the occurrence of an iron dyshomeostasis when dZIP13 is repressed. However, this iron dyshomeostasis could either be interpreted as a primary event or a secondary event. If this is the result of a primary event, dZIP13 might directly affect iron homeosatsis; if not or in the secondary scenario, dZIP13 might directly affect intracellular zinc distribution in such a way (without affecting the total zinc level) that results in iron dyshomeostasis. How to distinguish these two possibilities?

Defects of dZIP13–RNAi flies are greatly rescued by treating with iron but not zinc supplementation (Xiao et al., 2014). This favors “the iron defect as the primary event” explanation. However, this is not a compelling piece of evidence since it could be argued that extracellular zinc supplement would be hard to rescue intracellular zinc redistribution defect conceivably happening in the dZIP13–RNAi flies. In other words, it could be contrived that dZIP13 affects intracellular distribution which cannot be rescued by external zinc changes, but the iron consequence as a result of zinc defect in this case could be rescued more readily by iron.

Ferric staining of the gut and native gel showed iron failed to efficiently incorporate into ferritin when dZIP13 was knocked down (Xiao et al., 2014). This demonstrates that dZIP13 is involved in iron loading to ferritin. This constitutes a good but still not definite evidence to support dZIP13 as an iron transporter. It could be argued that zinc dyshomeostasis in the ER/Golgi may disrupt iron incorporation into the ferritin proteins. dZIP13 as an iron transporter is such an unexpected finding and so much against intuition, because we have to propose it not only as an iron transporter but an iron exporter (instead of zinc importer as most other ZIP proteins). In this way, we need to be extremely cautious to reach this conclusion and have to challenge us repeatedly for alternative possibilities. Before we go further we need to introduce briefly iron homeostasis in the fly.

## Iron homeostasis in *Drosophila* is distinct from that in mammals

The maintenance of iron homeostasis by cells involves iron uptake, utilization, storage, and excretion. Many proteins participate in these processes in mammalian organisms, including transferrin (Tsf), transferrin receptor (TfR1), duodenal cytochrome b (Dcytb), divalent metal-ion transporter 1 (DMT1), ferritin, mitoferrin (Mfrn), mitochondrial ferritin (MtFt), ferropotin (FPN), ceruloplasmin (Cp), hephaestin, hepcidin, and so on (Hentze et al., 2010; Tang and Zhou, 2013a). Many but not all mammalian iron genes have homologs in the fly (Mandilaras et al., 2013; Tang and Zhou, 2013a). For the purpose of this review, we will only briefly summarize what we know about fly iron homeostasis. Please refer to Tang and Zhou (2013a) and Mandilaras et al. (2013) for a more complete review of iron metabolism in the fly.

For iron uptake in the fly, a proposed *Drosophila* DMT1/NRAMP2 ortholog, Malvolio (Mvl) (Rodrigues et al., 1995; Folwell et al., 2006), resides in the anterior and posterior parts of the middle intestine, as well as in the malpighian tubules, brain, testis and fat body (Folwell et al., 2006), may be responsible for iron uptake from extracellular environment into cells (Southon et al., 2008; Bettedi et al., 2011; Tang and Zhou, 2013b). Genome studies also suggest that there are candidate sequences for Dcytb in *Drosophila melanogaster* (Verelst and Asard, 2003). There are also transferrin homologs in the fly genome (Yoshiga et al., 1999; Adams et al., 2000; Tiklová et al., 2010). Interestingly there is no obvious transferrin receptor in the genome (Dunkov and Georgieva, 2006).

For mammalian iron efflux, accompanied with iron loading to transferrin, intracellular Fe^2+^ is oxidized to Fe^3+^ under the action of the ferroxidases hephaestin (HEPH, intestinal, and CNS multicopper ferroxidase) or ceruloplasmin (CP, systemic multicopper ferroxidase; De Domenico et al., 2007). No obvious ferroportin homologs have been found in the *Drosophila* genome (Winzerling and Pham, 2006; Tang and Zhou, 2013a). However, four MCO candidate genes exist in the fly (Lang et al., 2012). Whether any one of these MCOs acts as ferroxidase is not clearly established yet.

*Fer1HCH* and *Fer2LCH* (Dunkov and Georgieva, 1999) encode the fly counterparts of mammalian ferritins. In contrast to mammalian cells, ferritin in *Drosophila* is not cytosolic, instead, it is secreted. Ferritin in the hemolymph is full of loaded iron. In *Drosophila* cells, intracellular iron would be incorporated into ferritin for storage and transporting via the ER/Golgi secretion path to the hemolymph and other cells (Tang and Zhou, 2013b). In this way, fly ferritin is responsible for dietary iron absorption and transport (Tang and Zhou, 2013b). Ferritin is retained in the ER or Golgi complex before secretion (Missirlis et al., 2007). ER/Golgi in the fly therefore turns out to be an iron-rich area and iron efflux via the secretory compartments is a key iron efflux pathway in this organism and likely many other insects (Xiao et al., 2014).

In comparison to the fly, in mammals, only a minor amount of ferritin (generally iron-poor) is found in the serum. As stated above, mammalian iron is exported by ferroportin and very little iron is moved across the secretory compartment. Nevertheless, the ER/Golgi still needs iron as some iron-containing enzymes are also stationed in the secretory pathway in mammals (Tang and Zhou, 2013b). In this context, there presumably should be an iron transporter on ER and/or Golgi membranes in both the fly and mammals.

## Further evidences for dZIP13 as an iron exporter

As mentioned, since different from that of mammalian organisms, *Drosophila* effluxes iron through the ER/Golgi secretory pathway via ferritin, a large amount of iron has to be loaded in the secretory pathway in the gut to satisfy the body iron demand. No previous candidates have been uncovered to fulfill this iron efflux role. dZIP13, surprisingly, is involved in the iron loading to ferritin. Does this mean dZIP13 relocates iron from the cytosol to the secretory pathway? Although that would be the most straight forward explanation, strictly speaking it is still not necessarily so. It could be contested that dZIP13 affects zinc homeostasis in the secretory pathway, which then affect iron loading to the ferritin. For example, zinc surplus in the ER/Golgi could cause stress, which affect iron loading to ferritin. Alternatively, excess zinc could compete with iron for ferritin binding so little iron is incorporated.

If zinc imbalance in the secretory pathway could so dramatically affect iron homeosatsis, we expect some other similarly resident proteins such as dZIP7 (Catsup) or dZnT7 (CG6672/ZnT86D) should also be involved in this process. Changing the expression of both these two genes by RNAi or overexpression leads to dissimilar phenotypes from those of altering dZIP13 expression, i.e., no obvious iron phenotypes in dZIP7- or dZnT7-modulated flies (Qin et al., 2013; Xiao et al., 2014). Genetic interaction studies of dZIP13 with dZIP7 or dZnT7 also reveal no significant effect of dZIP7 and dZnT7 on dZIP13 iron functions (Xiao et al., 2014). This supports strongly that the iron effects seen in dZIP13–RNAi flies are not due to zinc dyshomeostasis in the ER/Golgi compartments.

Direct evidence supporting dZIP13 as an iron exporter came from radioactive Fe transport assays (Xiao et al., 2014). dZIP13 expressed in *Escherichia coli* facilitated a linear time course of iron efflux. Isolated ER/Golgi from dZIP13–RNAi *Drosophila* larvae also exhibited reduced iron uptake rates whereas that from dZIP13–OE flies was significantly increased (Xiao et al., 2014). Taken together, all these evidences accumulatively indicate that dZIP13 is an iron exporter, and the iron phenotypes we saw in dZIP13–RNAi were indeed due to defective iron efflux.

## Iron exporting function of ZIP13 in mammals?

So far there is no direct evidence indicating that ZIP13 is an iron exporter in mammalian organisms. This is partially due to the fact that the possibility has rarely or probably never been explored. There remains a possibility that mammalian ZIP13 may function differently from dZIP13, although they share the greatest homology in protein sequence. We found when hZIP13 was expressed in the fly, phenotypes were reminiscent of, but much milder than, those of dZIP13 (Xiao et al., 2014). Defects of dZIP13–RNAi could be partially rescued by hZIP13 expressed in the fly (Xiao et al., 2014). This suggests that hZIP13 can function somewhat similarly to dZIP13 at least in the fly.

If ZIP13 supposedly exports iron, why no obvious iron phenotypes were observed in human SCD–EDS patients or rodent ZIP13–knockout (ZIP13–KO) models, as those in dZIP13–RNAi flies? This could be because in the fly the secretory pathway is loaded with iron, and the secretory pathway is the primary, if not the sole, iron exporting venue. In contrast in mammals, cellular iron is exported by plasma-membrane-resident ferroportin instead of the ER/Golgi path via secreted ferritin. Along this line of thinking, dZIP13 deficiency in the fly will result almost iron export shutdown, ending with overall iron absorption defect and deficiency. In mammals, iron is not exported via this avenue, so no general iron phenotype might be observed.

But iron is still needed in the mammalian ER/Golgi because some enzymes such as collagen crosslinking enzymes LH and PH4 need it as a cofactor. While it could be envisioned that the iron exporting function for *Drosophila* ZIP13 is highly demanding, that for mammalian ZIP13 is not so since much less iron is needed to pump into the secretory compartments in mammalian cells.

Overall, evolutionarily speaking, ZIP13 may also work as an iron exporter in mammalian cells to provide iron to ER/Golgi organelle. But direct evidences are still needed to support this assertion.

## ZIP13 as also a Zinc transporter?

Our studies in the fly indicate that dZIP13 is an iron exporter (Xiao et al., 2014). But does that mean dZIP13 has no zinc function? Probably not true. In the fly, dZIP13–RNAi phenotypes can be rescued by iron but not zinc. Nevertheless, we also noticed slightly altered zinc levels (Xiao et al., 2014), though we are not sure whether this is a primary or secondary event due to iron dyshomeostasis. In other studies, Fukada et al. found that ZIP13–KO mice show defects mimicking those of SCD–EDS patients (Fukada et al., 2008). Further studies showed that ZIP13 mainly expresses and functions in mesenchyme-originating cells, and locates in the Golgi apparatus in the mouse (Fukada et al., 2008). Zinc level in the Golgi was slightly increased in ZIP13–KO primary dermal fibroblasts as compared to that in wild-type cells, while zinc concentration in the cell nucleus was decreased (Fukada et al., 2008), suggesting that ZIP13 functions as a zinc transporter transporting zinc from the Golgi to the cytosol (Fukada et al., 2008). Consistent with being a zinc transporter, it was also reported that dietary zinc-deficiency up-regulates ZIP13 mRNA level in mice (Guo et al., 2011).

Biochemical characterization of human ZIP13 protein by Bin et al. demonstrated in cell culture studies that zinc importing activity of ZIP13, when assayed by metallothioneins (Mtns) expression or zinc probe FluoZin-3 after ZIP13 overexpression, is detectable when 100 μM zinc is added to the media (Bin et al., 2011). However, under normal growth conditions, overexpression of ZIP13 did not cause detectable zinc level changes (Bin et al., 2011). This suggests that ZIP13 is at least able to participate in zinc metabolism when zinc levels are high, but at normal conditions its zinc transporting efficiency might be less evident.

A research of zinc homeostasis and immune status in broiler chickens showed that both in jejunum and caecal tonsil, ZIP13 expression is the only measured zinc transporter impacted by dietary zinc source (Troche et al., 2015), suggesting that ZIP13 responds to zinc metabolism in chickens.

Another cell culture study by Jeong and his colleagues using antibody against native ZIP13 to determine the subcellular localization of endogenous ZIP13 indicated that ZIP13 localized to punctate vesicles dispersed throughout the cytoplasm (Jeong et al., 2012). To ascertain zinc uptake properties of Zip13, ZIP13 was overexpressed and a minor part was found mislocalized to the cell surface, enabling cellular zinc uptake assay resulted in zinc accumulation (Jeong et al., 2012). ZIP13-dependent zinc uptake was similar to other mammalian ZIP transporters and was not competed by excess extracellular iron (Jeong et al., 2012). These results indicate that ZIP13 imports zinc specifically. It is worthy of noting, however, that this experiment did not exclude iron transporting ability of ZIP13 for two reasons. The first is that zinc and iron may not necessarily compete for the same spot of transportation. Consistently, structure studies have found that ZIP may contain two metal binding sites (Zhang et al., 2017). More importantly, the competition experiment with extracellular iron would only address the iron importing function, but not the possible iron exporting function of human ZIP13, as claimed for dZIP13.

Considering all the evidences, zinc transporting of ZIP13 is entirely possible, although its zinc moving efficiency might be compromised compared to other ZIP transporters. This is consistent with the sequence data in that ZIP13 lacks a His-rich region in all its extracellular domains. According to biochemistry studies, the extracellular domains of ZIPs are likely involved in increasing transporting efficiency but not selective specificity (Jeong et al., 2012).

## The three current models to explain how ZIP13 mutations cause spondylocheirodysplasia-ehlers-danlos syndrome

As described before, collagen synthesis of SCD–EDS patients is abnormal in that crosslinking catalyzed by LH and PH4 is defective. During collagen synthesis, ER is the place where different collagen types are synthesized and modified (Myllyharju and Kivirikko, 2004). Among the various modifications, hydroxylation of collagen lysyl and prolyl residues is a highly conserved enzymatic process involving Fe^2+^ as the cofactor (Tuderman et al., 1977; Puistola et al., 1980). Externally supplied iron is needed for the enzymatic assays of the enzymes *in vitro* (Tuderman et al., 1977; Murad et al., 1985), suggesting iron is probably not tightly bound to these enzymes. On the other hand, zinc has been reported as an inhibitor of prolyl hydroxylase (Myllylä et al., 1977; Puistola et al., 1980).

**Figure 2:**
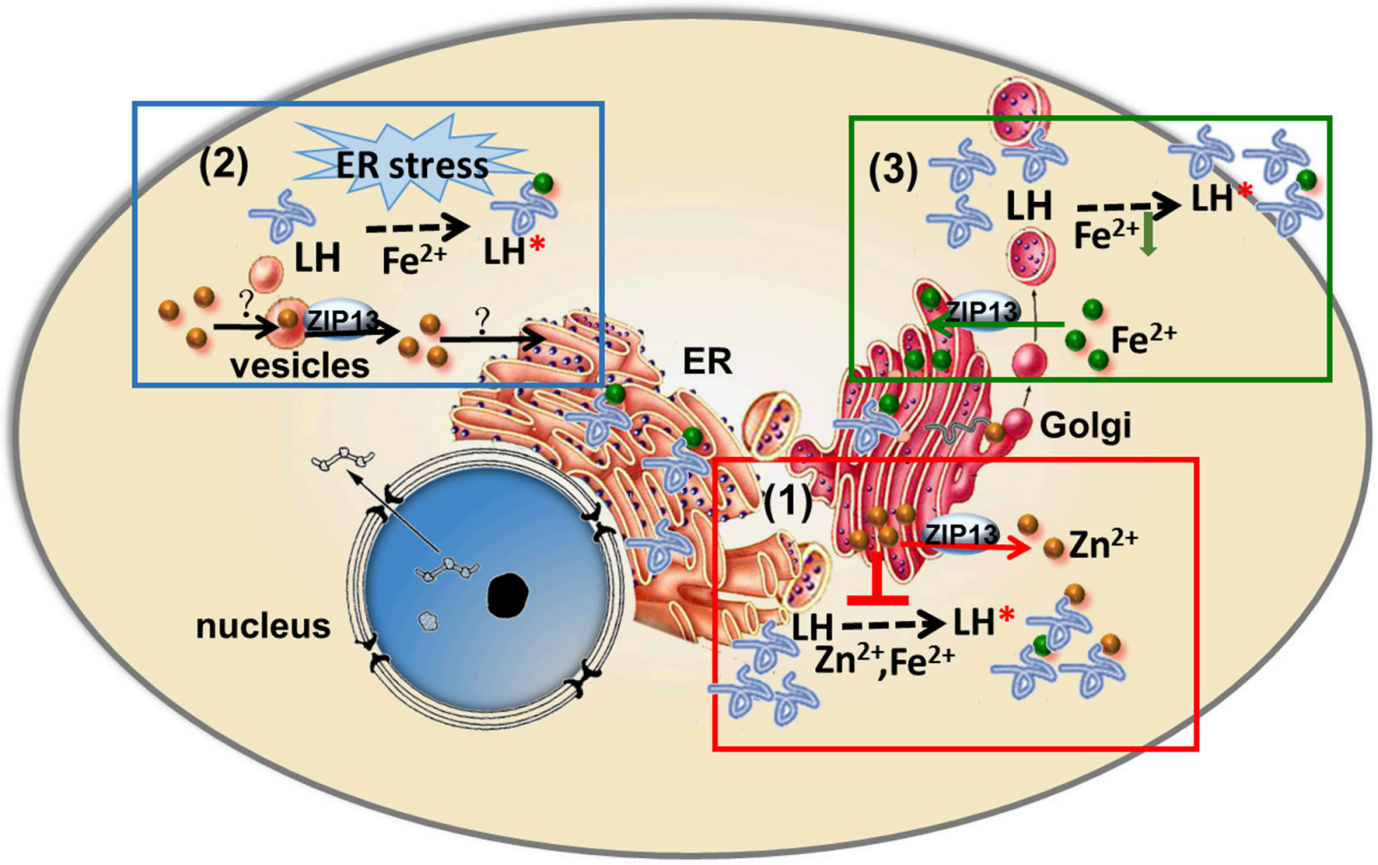
Three models of ZIP13's action proposed three entirely different etiology for SCD-EDS. (1) SCD-EDS is suggested to arise from increased zinc accumulation in the ER/Golgi. ZIP13 imports zinc from ER/Golgi to cytosol, so the absence of ZIP13 results in zinc accumulation in ER/Golgi. The accumulated zinc competes with iron for binding to LH and PH4, leading to disrupted biosynthesis of collagens and SCD-EDS. (2) SCD-EDS is due to zinc deficiency in the ER/Golgi. ZIP13 is responsible for zinc releasing from vesicular stores for use in the secretory pathway, so ZIP13 loss of function leads to general ER dysfunction and the pathogenesis of SCD-EDS. (3) SCD-EDS is caused by iron deficiency in the ER/Golgi. ZIP13 exports iron from cytosol into the secretory pathway, so ZIP13 loss of function results in iron deficiency in the ER/Golgi compartments, leading to collagen crosslinking defect and SCD-EDS.

Based on results from different studies as summarized in this review, three entirely different models have been proposed to explain the etiology of SCD–EDS. Since most ZIPs are involved in zinc transportations and the closest homolog of ZIP13 is ZIP7, which has been shown to be a Golgi-resident zinc importer, it is naturally to suggest that ZIP13 is responsible for zinc importing from ER/Golgi to cytosol in mouse and human cells (Figure 2; Fukada et al., 2008; Giunta et al., 2008; Bin et al., 2011; Jeong et al., 2012). Along this line, one model proposes that ZIP13 loss of function results in zinc accumulation in ER/Golgi, which competes with iron for binding to LH and PH4, two critical enzymes using iron as a cofactor in the secretory pathway for collagen hydroxylation (Figure 1; Myllyharju, 2008), leading to disruptive biosynthesis of collagens and SCD–EDS (Figure 2; Fukada et al., 2008; Giunta et al., 2008). Further supporting this, Fukada et al. found that zinc is slightly increased in the Golgi and decreased in the nucleus of ZIP13 KO cells (Fukada et al., 2008). They proposed that this zinc defect, somehow led to observed impairment in bone morphogenic protein (BMP) and TGF-β signaling pathway, and resulted in the nuclear shift of SMAD proteins and dysregulation of BMP/TGF-β-mediated genes expression critically involved in bone, tooth, and craniofacial skeletogenesis (Fukada et al., 2008).

In contrast, Jeong et al. based on their cellular localization of ZIP13 to the vesicular compartment, claimed that ZIP13 might function to release labile zinc from vesicular stores for use in the ER and other compartments (Jeong et al., 2012), and proposed that ZIP13 loss of function could lead to general ER dysfunction and thus explained the biochemical findings of SCD–EDS (Figure 2; Jeong et al., 2012).

The above two models of ZIP13's action proposed two entirely different etiologies for SCD–EDS (Figure 2). In the first model, SCD–EDS is suggested to arise from increased zinc accumulation in the ER/Golgi [Figure 2, box(1); Fukada et al., 2008; Bin et al., 2011], while the second proposed that SCD–EDS is due to zinc deficiency in the ER/Golgi [Figure 2, box (2); Jeong et al., 2012].

The finding of dZIP13 as an iron exporter offers a new and more straightforward explanation for SCD–EDS [Figure 2, box (3)]. If hZIP13, or for that matter mammalian ZIP13 altogether, also works as an iron exporter, this would be the only iron transporter so far identified in higher organisms in the ER/Golgi, providing the necessary ions needed in these compartments. In the absence of ZIP13, the secretory compartments will lack iron, a cofactor for the enzymatic activities of LH and PH4 (Figure 1), resulting in collagen crosslinking defect.

The baffling finding that in cells from SCD–EDS patients LH and PH4 are normal could also be well-explained by ZIP13 being as an iron exporter. LH and PH4 activities are assayed *in vitro* with a buffer containing ferrous iron. Because SCD–EDS patients are not defective in the proteins of LH and PH4 *per se*, what is happening is that these enzymes do not “see” iron *in vivo*, but *in vitro* when iron is supplemented normal activities of these enzymes would certainly be observed.

**Figure 3:**
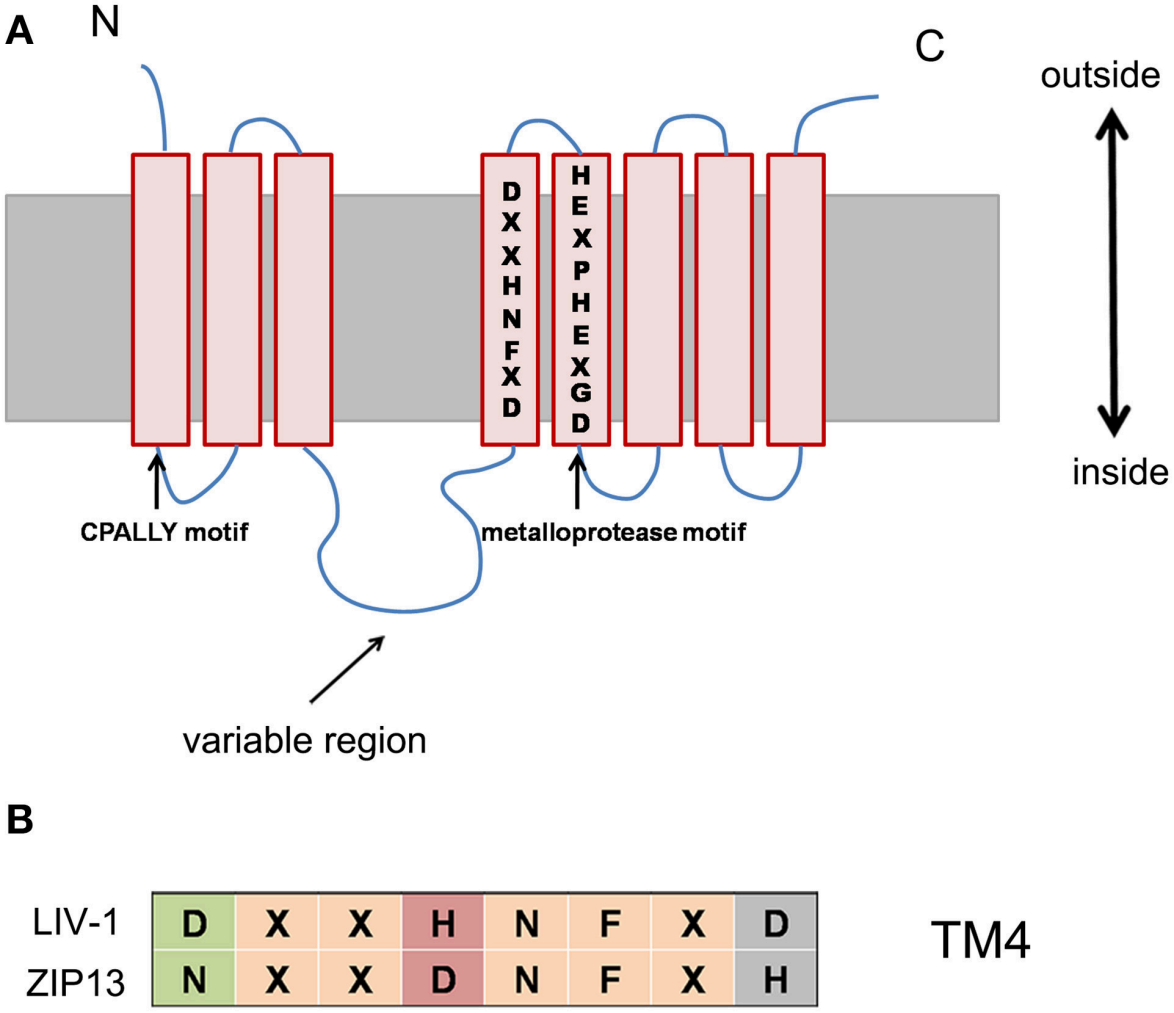
The conserved consensus sequence, DxxHNFxD in TM4 of LIV-1 subfamily, is changed to NxxDNFxH in ZIP13. **(A)** Schematic of conserved motif and sequence among the LIV-1 subfamily members. **(B)** This alignment demonstrating the highly conserved motif in TM 4 for the LIV-1 family and ZIP13.

If the new scenario is true or ZIP13 acts as an iron exporter, what unique features of ZIP13 might confer it such special transporting ability? The answer is largely unknown, however we can gain some insights from its sequence. The closest homolog of ZIP13 is ZIP7 (Jeong and Eide, 2013). But ZIP7 is an established zinc transporter moving zinc ion from Golgi to the cytosol (Huang et al., 2005). Sequence alignment of the LIV-1 subfamily members including human ZIP4, ZIP5, ZIP6, ZIP7, ZIP8, ZIP10, ZIP12, ZIP13, and ZIP14 shows that in addition to the five universally conserved residues found in all ZIP family members, most conserved residues within the LIV-1 subfamily are located in TM5 (Eide, 2004; Dempski, 2012). In addition, there are some conserved motifs among LIV-1 families, such as CPALLY motif and metalloprotease motif (Figure 3; Taylor et al., 2007). Notably, another conserved consensus sequence, DxxHNFxD in TM4 of LIV-1 subfamily, is changed to NxxDNFxH in ZIP13 (Figure 3; Xiao et al., 2014). The functional consequences of these sequence variations remain unknown and certainly worth exploring.

If SCD–EDS is indeed due to lack of iron in the ER/Golgi compartments, will iron supplement benefit these patients? We are not certain, but likely not very effective due to the following reasons. Firstly, alteration of iron levels within an organelle is normally less efficient than that in the cytosol. This is because it subjects to another level of control on top of that of the cytosol. In other words, it is a compartment within another compartment. Even when the cytosolic iron of a cell is altered, iron within an organelle of that cell may not be effectively changed. Mitochondria for example, have their own control of iron homeostasis on top of the other cellular iron control. Secondly, SCD–EDS mutations so far identified might be all null or close to null (Bin et al., 2014). In the absence of ZIP13 protein, it might be difficult to move iron into ER/Golgi even when the cytosolic iron level is higher. When we knocked-down dZIP13 expression in the fly, weaker collagen defects could also sometimes be observed. The collagen defects could be rescued by iron supplementation in the diet (Xiao et al., 2014). These flies, however, have partially functional dZIP13 due to incomplete suppression abilities of RNAi.

## ZIP family members with “unconventional” transporting properties

dZIP13 turns out to be very unusual in that it not only transports iron, but the transport is in the opposite direction! Is there any precedent for this?

In fact, the ZIP is short for Zrt-, Irt-like protein (Lye et al., 2012; Jeong and Eide, 2013; Xiao and Zhou, 2016), including many zinc and iron transporters. There are quite a few examples of ZIPs acting as iron transporters. *Arabidopsis thaliana* Irt1 and Irt2 (iron regulated transporters) were discovered by complementation of yeast zinc importer *zrt1 zrt2* mutant (Zhao and Eide, 1996). Considering that Irt1 and Irt2 are iron transporters, while Zrt1 and Zrt2 are zinc transporters, it seems that subtle changes in the amino acids or physiological context could have an important effect on the cation selectivity of ZIPs (Dempski, 2012).

In mammals, ZIPs were initially believed to be zinc importers. Recently, certain members of ZIPs have been reported to be capable of transporting iron and other metal ions. ZIP8 and ZIP14 (Liuzzi et al., 2006; Gao et al., 2008; Zhao et al., 2010; Coffey and Knutson, 2017) are two notable examples. Both ZIP8 and ZIP14 belong to the LIV-1 subfamily. Overexpression of mouse ZIP14 (mZIP14) increases cellular accumulation of iron and zinc in HEK 293H cells while suppression of ZIP14 expression decreased the uptake of iron and zinc by mouse hepatocytes (Liuzzi et al., 2006). Girijashanker et al. indicated that Cd^2+^ (apparent Km of 1.10 ± 0.02 μM) and Mn^2+^ (apparent Km of 18 ± 2 μM) can also be transported by ZIP14 while Zn^2+^ uptake by this protein could be inhibited by Cd^2+^, Mn^2+^, and Cu^2+^ (Girijashanker et al., 2008).

ZIP8 is expressed on the plasma membrane of cells such as the testis (Dalton et al., 2005). Function analysis of ZIP8 in MFF cells and *Xenopus laevis* oocytes demonstrated that ZIP8 have high affinity for Zn^2+^ and Cd^2+^, and mediates Cd^2+^ and Zn^2+^ uptake (He et al., 2006; Liu et al., 2008). Cadmium uptake is inhibited by Zn^2+^, Cu^2+^, Pb^2+^, and Hg^2+^, but not affected by Mn^2+^ or Fe^2+^ (Liu et al., 2008). The cell-surface expression of ZIP8 was enhanced by iron supplement in rat hepatoma cells (Wang et al., 2012), but the mechanism has not yet been elucidated.

Further study has demonstrated that, besides zinc and iron, many ZIP-family transporters can also transport cations such as copper, nickel, cadmium, and manganese (Dempski, 2012). For example, hZIP4, the gene whose mutations are involved in acrodermatitis enteropathica (AE) (Küry et al., 2002; Wang et al., 2002), was reported to transport copper and nickel in *X. laevis* oocytes (Antala and Dempski, 2012).

The above examples are all ZIPs transporting other metal ions in the inward direction. Is there any precedent that a ZIP member can export metal ions?

Bidirectional transport of zinc was first reported in *Saccharomyces cerevisiae* for the transporter Yke4p, encoded by yeast YIL023C (YKE4), which is the ortholog of mouse Ke4 (Kumánovics et al., 2006). Yke4p is a novel member of ZIP family localized to the ER membrane (Kumánovics et al., 2006). In high zinc medium, Yke4p eliminates the cytosolic zinc into the secretory pathway to avoid toxic zinc accumulation in the cytosol whereas under low cytosolic zinc conditions, Yke4p relocates zinc from the secretory pathway to the cytosol (Kumánovics et al., 2006). Current genetic and biochemical evidences indicate that Yke4p is capable of transporting zinc both into and out of the vesicular apparatus, depending on the zinc status in the cytosol (Kumánovics et al., 2006).

Outside of the ZIP family, a member of ZnT family is also capable of bidirectional zinc transport (Cragg et al., 2002; Valentine et al., 2007). Valentine et al. showed that human ZnT5 variant B [ZnT5B (hZTL1)], an isoform of ZnT5 expressed at the plasma membrane, operates in both the uptake and the efflux directions when expressed in *X. laevis* oocytes (Cragg et al., 2002; Valentine et al., 2007). They found that ZnT5B is expressed at the plasma membrane in Caco-2 cells and *X. laevis* oocytes, and can mediate zinc efflux against a concentration gradient but can also function in the opposite direction in the same system. Because dramatically elevated total intracellular zinc concentration was observed in Caco-2 cells and oocytes expressing ZnT5B at the plasma membrane, while zinc sequestration into specific intracellular compartments was not affected, ZnT5B mediating zinc uptake across the plasma membrane was suggested (Cragg et al., 2002; Valentine et al., 2007). These data demonstrate that ZnT5 can not only operate in an efflux mode, but also has the capability to mediate zinc uptake.

Besides zinc transporters, many ion exchangers move different ions in opposite directions. Na^+^/Ca^2+^ exchanger (NCX), found in cardiac muscle cells and elsewhere in the body, transports three Na^+^ ions into the cell in exchange for one Ca^2+^ ion transported out of the cell (Liao et al., 2012). The glucose transporter can exist in different conformations that expose the glucose binding site to either the extracellular fluid or the cytosol to ensure the counter transport of glucose in cells (Thorens and Mueckler, 2010). Glucose is transported from the extracellular fluid into the cytosol by transporters when cells need to uptake glucose for energy source. However, the direction of transport will be opposite if the glucose concentration is higher inside the cell than outside (Medina and Owen, 2002; Thorens and Mueckler, 2010).

## Conclusions

*Drosophila melanogaster* has served as a good research platform to investigate the function of metal transporters and studies in this organism may advance our understanding of metal homeostasis and its relevance to human disease(Xiao et al., 2013). With the help of this platform, we identified that the primary role of dZIP13 is exporting iron from the cytosol into the secretory pathway. On top of the existing models, we suggest a more straightforward model to explain the pathogenesis of SCD–EDS; the activities of critical enzymes for collagen synthesis in these patients are affected due to iron deficiency in the secretary compartments. A critical issue remains to be solved is whether mammalian ZIP13 is also responsible to provide iron to the secretory compartments for collagen synthesis.

## Author contributions

GX drafted the initial version of the manuscript; BZ drastically revised the manuscript. All authors made intellectual contributions, edited, and approved the manuscript for publication.

### Conflict of interest statement

The authors declare that the research was conducted in the absence of any commercial or financial relationships that could be construed as a potential conflict of interest.
